# High Mobility Group Box 1 Contributes to the Acute Rejection of Liver Allografts by Activating Dendritic Cells

**DOI:** 10.3389/fimmu.2021.679398

**Published:** 2021-06-10

**Authors:** Yi Chen, Wenmin Zhang, Hui Bao, Wubing He, Lihong Chen

**Affiliations:** ^1^ Department of Pathology, The School of Basic Medical Sciences, Fujian Medical University, Fuzhou, China; ^2^ Department of Pathology, Mengchao Hepatobiliary Hospital of Fujian Medical University, Fuzhou, China; ^3^ Institute of Oncology, Fujian Medical University, Fuzhou, China; ^4^ Diagnostic Pathology Center, Fujian Medical University, Fuzhou, China; ^5^ Department of Emergency, Fujian Provincial Hospital; Shengli Clinical Medical College of Fujian Medical University, Fuzhou, China

**Keywords:** dendritic cells, glycyrrhizic acid, HMGB1, acute rejection, liver transplantation

## Abstract

Acute rejection induced by the recognition of donor alloantigens by recipient T cells leads to graft failure in liver transplant recipients. The role of high mobility group box 1 (HMGB1), an inflammatory mediator, in the acute allograft rejection of liver transplants is unknown. Here, rat orthotopic liver transplantation was successfully established to analyze the expression pattern of HMGB1 in liver allografts and its potential role in promoting the maturation of dendritic cells (DCs) to promote T cell proliferation and differentiation. Five and 10 days after transplantation, allografts showed a marked upregulation of HMGB1 expression accompanied by elevated levels of serum transaminase and CD3^+^ and CD86^+^ inflammatory cell infiltration. Furthermore, *in vitro* experiments showed HMGB1 increased the expressions of co-stimulatory molecules (CD80, CD83, and MHC class II) on bone marrow-derived DCs. HMGB1-pulsed DCs induced naive CD4^+^ T cells to differentiate to Th1 and Th17 subsets secreting IFN-γ and IL-17, respectively. Further *in vivo* experiments confirmed the administration of glycyrrhizic acid, a natural HMGB1 inhibitor, during donor liver preservation had therapeutic effects by reducing inflammation and hepatocyte damage reflected by a decline in serum transaminase and prolonged allograft survival time. These results suggest the involvement of HMBG1 in acute liver allograft rejection and that it might be a candidate therapeutic target to avoid acute rejection after liver transplantation.

## Introduction

Liver transplantation is an optional therapeutic choice for patients with end-stage liver disease (1, 2). Although surgical techniques and immunosuppressive drugs have been greatly improved, acute rejection after liver transplantation remains a major obstacle for long-term graft survival (3, 4). Currently, systemic immunosuppressive drugs including corticosteroids and mycophenolic acid are widely used before or after liver transplantation and have achieved good clinical effects (5). However, the nonspecific inhibition of the systemic immune system can cause serious side effects including infection, malignancy, and other drug-specific complications (6). Therefore, efforts should be taken to establish new methods that avoid acute rejection and allow the long-term viability of liver grafts.

Dendritic cells (DCs) are professional antigen presenting cells that participate in liver allograft rejection by presenting alloantigens to recipient-derived naïve T cells by direct, indirect, and semi-direct pathways (7, 8). In the direct pathway, donor-derived DCs transferred into the recipient as passengers in the portal tracts and hepatic veins of a liver graft migrate to recipient lymphoid tissues and induce T cell proliferation and differentiation (9). The indirect pathway refers to the biological process whereby recipient DCs take up, process, and present alloantigens on recipient MHC molecules to host T cells (9, 10). In the semi-indirect pathway, recipient DCs interact with intact donor MHC molecules on their surface *via* direct cell–cell interactions or the fusion of exosomes derived from donor DCs, which subsequently trigger T cell activation (11, 12). Successfully activated recipient naïve T cells can mobilize into the graft liver and differentiate into Th1 or Th17 subsets characterized by the secretion of IL-2 or IFN-γ, and IL-17, respectively (12). Th1 cytokines including IL-2 and IFN-γ enhance immune injury by recruiting more inflammatory cells into the liver grafts, whilst IL-17 impairs tolerance and recruits neutrophils (12, 13). Furthermore, alloreactive T cells generate CD8^+^ T cell responses and mediate graft tissue injury, causing damage to the biliary epithelium, hepatocytes, and endothelium cells (12). Many damage-associated molecular pattern molecules (DAMPs) released from damaged hepatocytes and liver sinusoidal endothelial cells promote cross-talk between DCs and alloreactive T cells, which induce inflammation after liver transplantation (14, 15).

High-mobility group box 1 (HMGB1), a damage-associated molecular pattern (DAMP), is expressed in the cell nucleus and facilitates DNA binding to regulate gene transcription in the normal physiological environment (16, 17). When tissues and organs are damaged, HMGB1 is released extracellularly from necrotic or damaged cells and initiates inflammatory cascades by triggering the secretion of cytokines, chemokines, and adhesion molecules, which promote leukocyte recruitment and infiltration (16, 17). Previous studies of the mechanism of acute and chronic rejection after heart transplantation revealed that the upregulation of HMGB1 expression was accompanied by inflammatory infiltration and that the blockade of HMGB1 prolonged liver allograft survival time (18). Furthermore, HMGB1 was critical for the early loss of transplanted islet cells in rats (19). However, the expression pattern of HMGB1 and its potential role in the acute rejection of liver transplants are unclear.

In this study, we detected the expression of HMGB1 in an MHC class II (MHC II)-mismatched rat liver transplantation model and found that the elevated expression of HMGB1 was accompanied by the infiltration of CD86^+^ and CD3^+^ cells. Our *in vitro* studies revealed that DCs treated with HMGB1 promoted T cell proliferation and differentiation. Blockade of HMGB1 *in vivo* by glycyrrhizic acid, a specific HMGB1 inhibitor, had therapeutic effects on the acute rejection of liver transplants, indicating HMGB1 might be a therapeutic target for the prevention of liver allograft rejection.

## Materials and Methods

### Animals

Specific pathogen-free 6-8-week-old Lewis and Brown Norway (BN) rats weighing 250-300 g were purchased from the Laboratory Animal Center of Fujian Medical University (Fujian, China). All rats were housed under specific pathogen-free conditions with a constant temperature (20 ± 2°C) and humidity (50 ± 10%). All animal protocols and husbandry were approved by the Ethics Committee of Fujian Medical University (No. 2015-29) and were in accordance with the NIH Guidelines for the Care and Use of Laboratory Animals.

### Liver Transplantation

Orthotopic liver transplantation with an improved two-cuff method was performed using a microsurgical technique as described previously (20, 21). In the allogeneic group, Lewis rats were used as donors and BN rats were used as recipients, whereas in the syngeneic group, the donors and recipients were both Lewis rats. In the sham group, Lewis rats only received the opening and closure of the abdomen under anesthesia for 35 min, which was the mean operation time of liver transplantation in recipient rats during surgery.

### Hematoxylin and Eosin (H&E) Staining, Immunohistochemistry (IHC), and Rejection Activity Index (RAI) Scores of Liver Grafts in Recipient Rats

According to previous experiments, acute rejection-induced injury occurs from approximately 5 d post surgery and becomes more severe with time. Thus, in this study, we focused on pathological changes at 5 and 10 d after transplantation. Three recipient rats from each group were randomly chosen at 5 and 10 d after transplantation and sacrificed by an overdose of sodium pentobarbital anesthesia followed by the collection of grafted livers and serum. The liver tissues were fixed in 4% paraformaldehyde and then embedded in paraffin. Midsagittal serial sections (4-μm thick) were cut and stained with H&E. The rejection activity index (RAI) of grafted livers was quantified with reference to the histopathology of the portal area, damage of the bile duct, and venous endothelial inflammation as described previously (22). To identify the infiltrating inflammatory cells, immunohistochemical analysis was performed. Paraffin sections of liver grafts were probed with rabbit monoclonal anti-CD3 antibody (1:500; ab86883, Abcam, USA) or monoclonal anti-CD86 antibody (1:200; ab239075, Abcam) overnight at 4°C. Then, the section was stained using the Streptavidin/Peroxidase Histostain™ Plus Kit (ZSGQ-BIO, Beijing, China) according to the manufacturer’s instructions. CD3^+^ and CD86^+^ cells per field were analyzed and calculated with Image pro plus software 6.0 (USA).

### Matrix-Assisted Laser Desorption/Ionization Time-of-Flight (MALDI-TOF) Profiling and Statistical Analysis

Proteins in graft liver tissues from the syngeneic and allogeneic groups were extracted and quantified using the BCA method. Then, 5 µg of protein was added into the MALDI target plate, dried, and analyzed by mass spectrographic analysis with a MALDI-TOF mass spectrometer (Bruker Microflex LRF, Bremen, Germany) under the linear positive mode. Raw data were obtained using Flexanalysis (Bruker, Bremen, Germany) and processed with the R packages MALDIquant and MALDIquantForeign against the RattusNorvegicus database. A threshold fold change > 1.2 or < 0.83 and a *p*-value < 0.05 was set to identify differentially-expressed proteins, which were shown in a volcano plot and heat map.

### Western Blotting Analysis

Total protein from liver tissues obtained from recipient rats was extracted by RIPA Lysis and Extraction Buffer (Thermo, USA) containing a protease inhibitor. After the protein concentration was measured by BCA kit, total protein was separated by 10% SDS-PAGE electrophoresis and then transferred to a nitrocellulose membrane. The membrane was probed with rabbit anti-HMGB1 antibody (1:10 000; ab18256, Abcam, USA) followed by incubation with horseradish peroxidase (HRP)-labeled secondary antibodies. Proteins were visualized with a chemiluminescence substrate kit (Pierce Biotechnology, Rockford, IL, USA). GAPDH was used as an internal control.

### RNA Extraction, cDNA Synthesis, and Quantitative Real-Time PCR

Total RNA was extracted using the QIAGEN RNeasy Mini Kit (Qiagen; Germany). Then, total RNA was synthesized with the PrimeScript 1st Strand cDNA Synthesis Kit (Takara, Japan) and quantitative real-time PCR assay was performed using TransStrat Top Green qPCR SuperMix (TransGen, Beijing, China). Primer sequences were as follows: HMGB1 gene, 5′-AGTTCAAGGACCCCAATGCC-3′ (forward) and 5′-TACTTCTCCTTCAGCTTGGCG-3′ (reverse); and GAPDH gene (internal expression control) 5′-TGTGAACGGATTTGGCCGTA-3′ (forward) and 5′-GATGGTGATGGGTTTCCCGT-3′ (reverse). The results of the threshold cycle (Ct) were analyzed using the 2^−ΔΔCt^ method after being normalized to the house-keeping gene GAPDH. The fold change in HMGB1 gene expression in the syngeneic and allogeneic groups was calculated relative to that in the sham group at 5 d post surgery.

### Detection of ALT and AST in the Serum of Recipient Rats

To obtain recipient rat serum, blood was collected from the oculomotor sinus of rats under ether anesthesia. After the blood had clotted in serum separator tubes for 4 h at room temperature, it was centrifuged at 1,000 ×g for 30 min to obtain serum. Serum levels of ALT and AST were measured by a commercial kit (Nanjing Jiancheng, Nanjing, China).

### Separation and Cultivation of Rat Bone Marrow-Derived Dendritic Cells

DCs were generated from rat bone marrow cells according to a previous protocol with minor modifications (23). Briefly, hind limbs were gently rinsed with PBS to collect bone marrow cells. After removing red blood cells, the remaining cells were cultivated in RPMI 1640 medium (Hyclone, USA) containing 10% fetal bovine serum (FBS; Thermo Fisher, USA) and penicillin/streptomycin at 37°C in a humidified atmosphere with 5% CO_2_ for 3 h. Subsequently, the adherent cells were re-suspended and cultured in RPMI 1640 medium with 20 ng/ml recombinant GM-CSF and 10 ng/ml IL-4 (PeproTech, USA) for 7 days. The cell culture medium was refreshed on days 3 and 5. On day 7 of cultivation, immature DCs were collected for experiments.

### Dendritic Cell Activation and Detection

Immature DCs were obtained from rat bone marrow cells as described above. To evaluate the effects of HMGB1 on the phenotype of DCs, immature DCs were stimulated with PBS (negative control), 1 µg/ml LPS (positive control), or 5 µg/ml HMGB1. After stimulation for 7 d, the cells were suspended and stained with FITC-conjugated mAbs to major histocompatibility complex II (MHC II), FITC-conjugated mAbs to CD80, and PE-conjugated mAbs to CD86 (BD Biosciences, CA, USA). Finally, the expression of markers on dendritic cells was detected by flow cytometry (BD Biosciences, CA, USA).

### Co-Cultivation of HMGB1-Stimulated DCs With Naïve CD4^+^ T Cells

To reveal whether HMGB1-stimulated DCs activated naïve CD4^+^ T cells, DCs were stimulated with HMGB1 for 48 h and then co-cultivated with naïve CD4^+^ T cells derived from the spleens of rats by magnetic-activated cell sorting (MACS) with a CD4^+^ T cell isolation kit (Miltenyi Biotec, Germany). Overall, 5 × 10^5^ dendritic cells stimulated with PBS, HMGB1, or LPS were seeded in six-well plates and then naïve CD4^+^ T cells labeled with 5- and 6-carboxyfluorescein diacetate succinimidyl ester (CFSE) (eBioscience, San Diego, CA, USA) were added. Then, 5 μg/ml Concanavalin A (Con-A) (Sigma-Aldrich, St. Louis, MO, USA) was used as a nonspecific stimulator of T cell proliferation, which was measured by flow cytometry (BD Biosciences, CA, USA).

### Cytokine Profile of T Cell Responses Stimulated by HMGB1 *In Vivo*

To eliminate the interference of dendritic cells when detecting the cytokine profile of T cells, intracellular cytokine staining of T cells was performed. Overall, 5.0×10^4^ DCs stimulated as described above were co-cultivated with 5.0×10^5^ naïve CD4^+^ T cells in 24-well plates containing 0.7 μL/mL GolgiStop (BD Biosciences, USA), 50 ng/ml phorbol 12-myristate 13-acetate (PMA) (Sigma, USA) and 750 ng/ml ionomycin (Sigma, USA) for 8 h at 37°C. The cells were suspended and labeled with anti-CD4-FITC on ice to distinguish T cells. Finally, cells were resuspended with 250 μL Fixation/Permeabilization buffer (BD Biosciences, Cytofix/Cytoperm kit, USA), and intracellular cytokine staining was detected by anti-rat IFN-γ-APC (ab239425, BioLegend, USA) and anti-rat IL-17-PE (ab186713, BioLegend). Finally, cell suspensions were analyzed by flow cytometry.

### Assessment of the Therapeutic Effects of Glycyrrhizic Acid on Liver Transplantation

To identify whether the blockade of HMGB1 eased acute rejection-induced liver injury after transplantation, a natural inhibitor of HMGB1 named glycyrrhizic acid (Sigma, USA) was used to treat the liver graft before transplantation. Before liver grafts in the treatment group were excised, livers were infused with 10 ml ice-cold Ringer’s solution containing 100 units heparin and 200 mg/ml glycyrrhizin (diluted in DMSO) under low pressure. Then, the graft was stored in Ringer’s solution containing 200 mg/ml glycyrrhizic acid at 4°C until transplantation. The perfusion and conservation fluid of grafts in the control group was Ringer’s solution containing DMSO. At 5 and 10 d post transplantation, H&E staining, and serum ALT and ALT levels were chosen to evaluate the severity of acute rejection as described above. Finally, six rats in each group were used for survival analysis.

### Statistical Analysis

The results are presented as the mean ± standard deviation and analyzed by GraphPad Prism version 6.0 software (GraphPad Software, San Diego, CA, USA). The difference among groups was determined by one-way analysis of variance (ANOVA) followed by Tukey’s post-hoc test. Statistical differences between two groups were determined by the Student’s *t*-test. Recipient rat survival data were determined using the Kaplan-Meier method and differences in survival time were tested by the Mantel-Cox log-rank method. *p* < 0.05 was considered statistically significant.

## Results

### Graft Histopathology of Liver Tissues and Inflammatory Cell Infiltration in Recipient Rats

**Figure 1:**
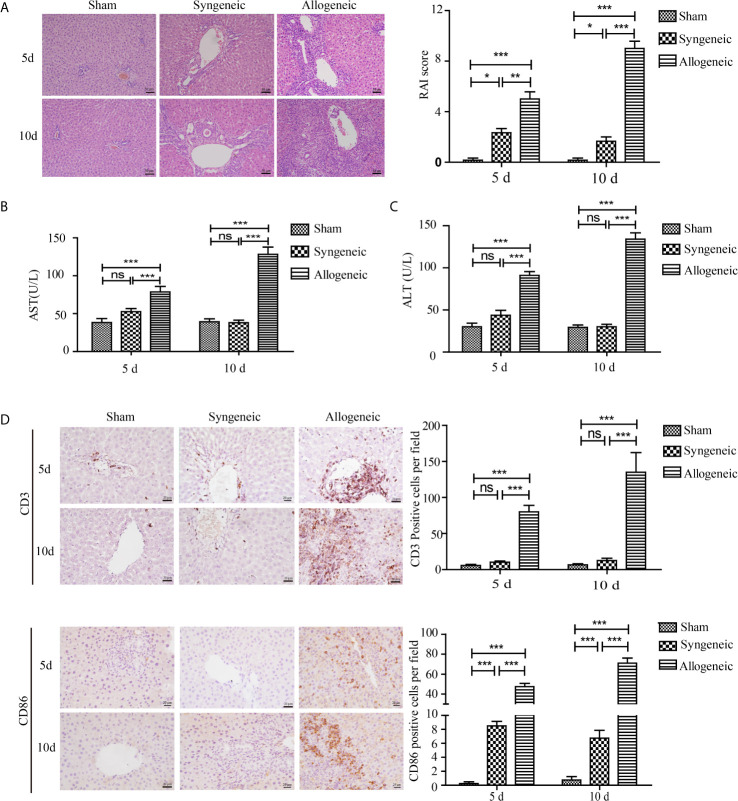
Establishment of a rat liver transplantation model and inflammatory cell infiltration in liver grafts. Liver grafts retrieved from Lewis rats were orthotopically transplanted into BN rats or Lewis rats (syngeneic and allogeneic groups respectively). The sham group received only the opening and closing of the abdomen. **(A)** Representative H&E staining of liver grafts (magnification, ×200). The right panel shows the RAI scores of the three groups. Severe parenchymal necrosis and destruction of bile ducts were present in the allogeneic group compared with the allogeneic and sham groups. **(B, C)** Serum concentrations of AST and ALT in recipient rats. Serum AST and ALT levels in the allogeneic group were highest among the three groups. **(D)** CD3^+^ and CD86^+^ inflammatory cells in liver grafts measured by IHC (magnification, ×200). The numbers of CD3^+^ and CD86^+^ cells per field in liver grafts were counted with Image pro plus and shown in the right panel. Marked infiltration of CD3^+^ and CD86^+^ inflammatory cells was observed in the allogeneic group, whereas the allogeneic and sham groups had few CD3+ and CD86+ inflammatory cells. All experiments were performed three times and data are presented as the mean ± SD. *p < 0.05, **p < 0.01, ***p < 0.001; ns, not significant.

The histopathological changes of livers transplanted orthotopically in an allogeneic milieu (Lewis rat liver implanted into BN or Lewis rats) at 5 and 10 d after transplantation are shown in **Figure 1A**. The liver tissues of the allogeneic group exhibited severe portal inflammation, endotheliitis, destruction of bile ducts, and mixed inflammatory cells infiltration compared with the syngeneic group, and the changes were in accordance with the RAI scores of the three groups. Similarly, serum levels of ALT and AST were highest in the allogeneic group at 5 and 10 d after transplantation compared with the other groups (**Figures 1B, C**). To identify the types of infiltrating inflammatory cells, cells were immunohistochemically stained for CD3 (a T cell surface marker) and CD86 (a DC surface marker) (**Figure 1D**). The allograft group had the highest number of CD3^+^ and CD86^+^ cells in the liver portal areas compared with controls (*p* < 0.05).

### MALDI-TOF Profiling Results Obtained From Syngeneic Liver Grafts and Allogeneic Liver Grafts

**Figure 2:**
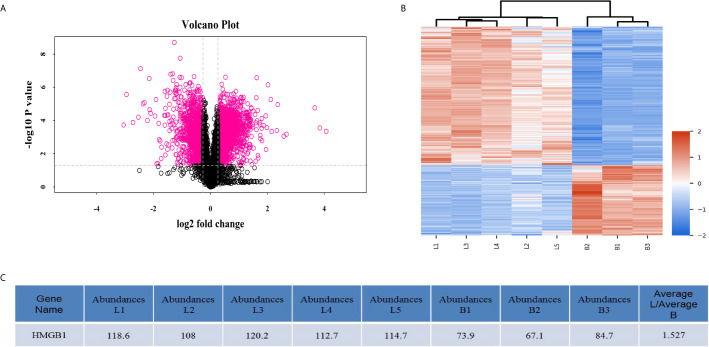
MALDI-TOF profiling data obtained from three syngeneic liver grafts and five allogeneic liver grafts. **(A)** Volcano plots of differentially-expressed proteins. The x-axis represents the negative log (base 10) of the adjusted p-value and the y-axis represents the log (base 2) of fold change. The dots in pink denote differentially-expressed proteins. Overall, 3276 differentially-expressed proteins including HMGB1 were identified by mass spectrometry. **(B)** Heatmap plots of the differentially-expressed proteins. The horizontal axis represents the samples and the upper horizontal axis represents the sample clusters. The vertical axis represents the differentially-expressed proteins and the left vertical axis shows the clusters of differentially-expressed proteins. L1–5 represents the five allogeneic liver grafts and B1–3 represents the three syngeneic liver grafts. Red represents upregulated proteins and blue represents downregulated genes. **(C)** HMGB1 expression was elevated in the allogeneic liver grafts.

Overall, 3276 differentially-expressed proteins were identified between the syngeneic and allogeneic groups (**Figure 2**). Among the differentially-expressed proteins, HMGB1 in the allogeneic group was expressed at a higher level than in the syngeneic group with a log2 fold change of 1.527, indicating the elevated level of HMGB1 might contribute to the acute rejection of liver transplants.

### Expression of HMGB1 Protein and mRNA in the Liver Tissues of Recipient Rats

**Figure 3:**
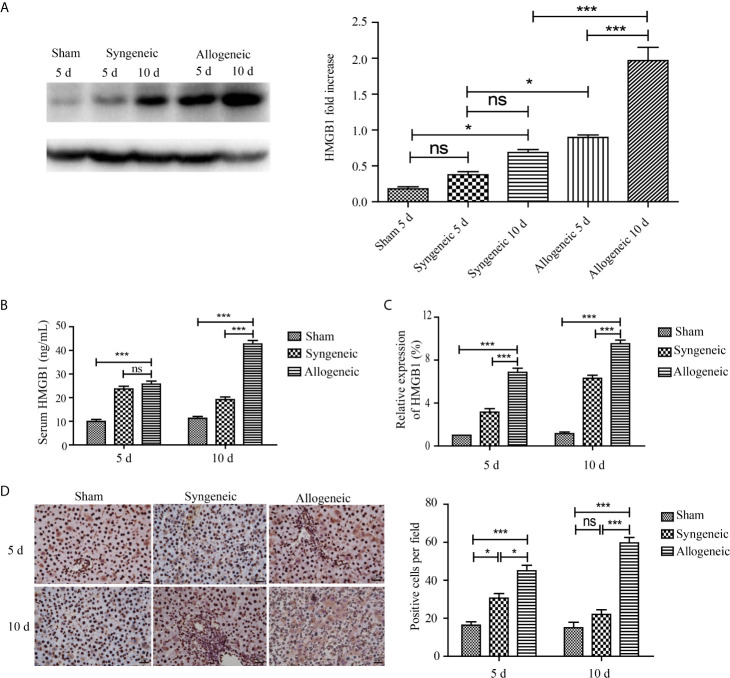
The expression of HMGB1 in liver grafts. After liver graft tissues and serum samples were collected from recipient rats at 5 and 10 d after transplantation, the HMGB1 mRNA and protein levels were measured by ELISA, qRT-PCR, and IHC. **(A)** Expression of HMGB1 protein in liver grafts tissues was evaluated by western blot and GAPDH was used as a control. HMGB1 in the allogeneic and syngeneic groups was increased with time, and its expression in the allogeneic group was higher than that in the syngeneic group at all timepoints post surgery. **(B, C)** HMGB1 mRNA and protein levels detected by ELISA and qRT-PCR. HMGB1 mRNA and protein levels in the allogeneic group were highest among the three groups. **(D)** HMGB1 protein detected by immunohistochemistry (magnification, ×400). HMGB1 positive cells were counted with Image pro plus and are shown in the right panel. Numbers of nuclear- and cytoplasm-positive cells were highest in the allogeneic group at 5 and 10 d after transplantation compared with the other groups. All experiments were performed three times and data are presented as the mean ± SD. *p < 0.05, ***p < 0.001; ns, not significant.

To measure the expression of HMGB1 protein and mRNA in recipient rats, liver graft tissues and serum samples were collected from recipient rats at 5 and 10 d after transplantation. The western blot results showed HMGB1 protein levels were higher in the allogeneic group than the other groups at 10 d post transplantation, and this tendency continued and was associated with the development of acute rejection (**Figure 3A**). However, no obvious difference in HMGB1 protein levels was found between the allogeneic and sham groups. Similar to the western blot results, the highest serum HMGB1 concentration and mRNA level in the allogeneic group were observed at 10 d after transplantation, both of which were higher than that in the other two groups (**Figures 3B, C**). Regarding the expression characteristics of HMGB1 in liver grafts, HMGB1 positive cells were mainly present in the nuclei of hepatocytes in the sham and syngeneic groups. In the allogeneic group at 5 and 10 d after transplantation, HMGB1 was present in the nuclei and cytoplasm of hepatocytes. Furthermore, the allogeneic group had the highest numbers of cells with nuclear and cytoplasmic HMGB1 staining (**Figure 3D**).

### HMGB1 Promotes the Maturation of DCs *In Vitro*

**Figure 4:**
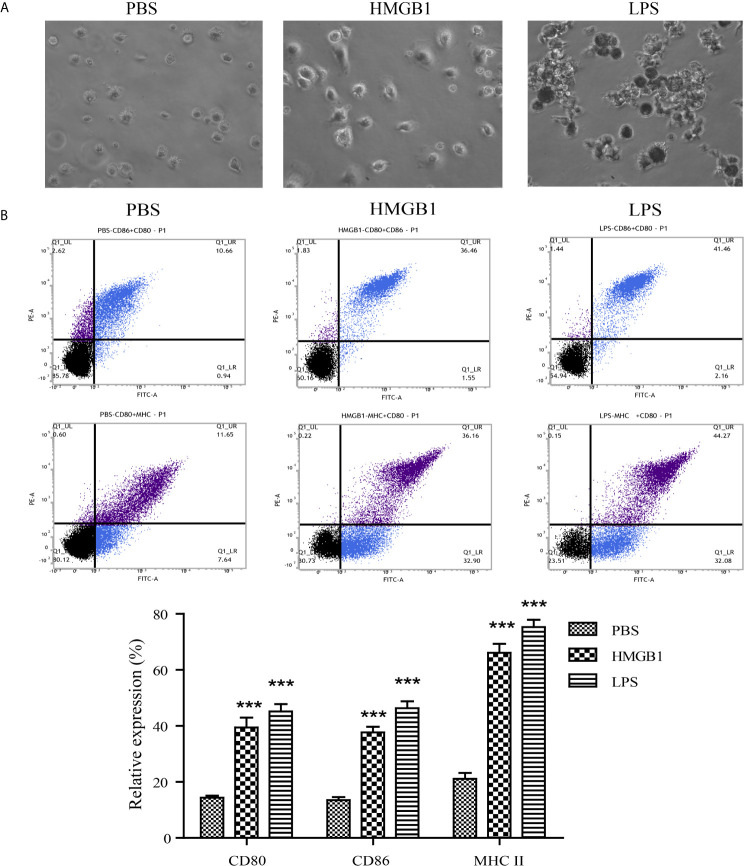
Features and expression of surface markers on DCs stimulated with HMGB1. DCs were stimulated with PBS, HMGB1, or LPS and the surface expressions of CD80, CD86, and MHC II were detected by flow cytometry. **(A)** Features of DCs stimulated with PBS, HMGB1, or LPS (magnification, ×400). Cultured DCs had irregular protrusions on their surfaces, especially the HMGB1- or LPS-pulsed DCs. **(B)** The percentage of surface marker expression on DCs stimulated with various antigens. HMGB1- or LPS-pulsed DCs had a higher expression of CD80, CD86, and MHC II compared with the PBS control. The lower panel shows the means ± standard deviations of the results from three individual experiments. ***p < 0.001 compared with the PBS control.

To determine the effects of HMGB1 on DCs *in vitro*, DCs were pulsed with PBS, LPS, or HMGB1. The features of DCs stimulated with PBS, HMGB1, or LPS analyzed by microscopy are shown in **Figure 4A**. Villi-like structures were observed on the surface of DCs. FACS data revealed that HMGB1 and LPS (positive control) upregulated the expressions of CD80, CD86, and MHC II on DCs compared with the PBS control (*p* < 0.05), as shown in **Figure 4B**. These results demonstrated that HMGB1 promoted the maturation of DCs.

### HMGB1-Stimulated DCs Activate Naïve CD4^+^ T Cells

**Figure 5:**
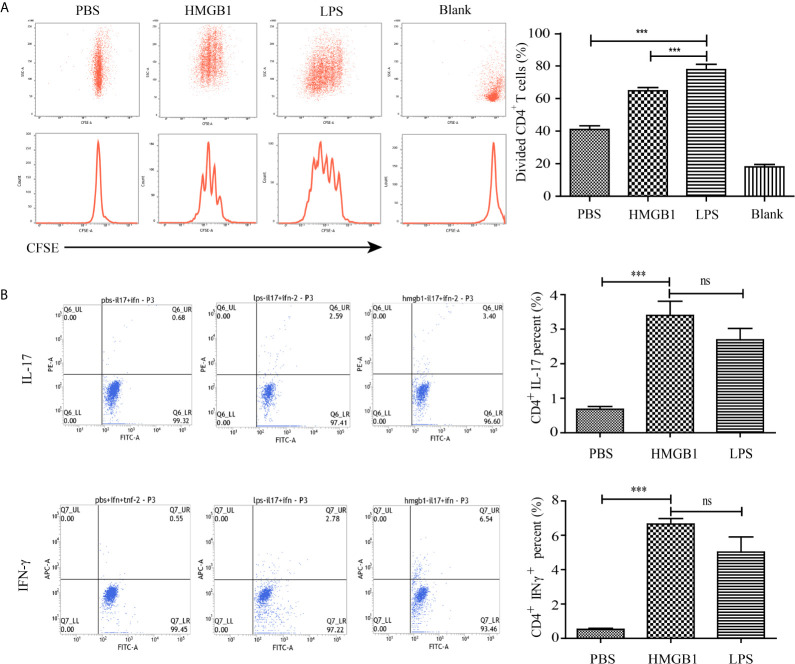
Proliferation and cytokine secretion of CD4+ T cells co-incubated with HMGB1-stimulated DCs. CD4+ T cells were extracted from the spleens of rats and incubated with HMGB1, LPS, or PBS-treated DCs. **(A)** The proliferation of co-incubated T cells was measured by CFSE labeling. The % proliferation of T cells pulsed by HMGB1-stimulated DCs was higher than those stimulated with control DCs. **(B)** Representative staining and percentages of IFN-γ- and IL-17-producing CD4+ T cells evaluated by flow cytometry. Data are expressed as the mean ± SEM from three independent experiments. ***p < 0.001; ns, not significant.

To assess whether HMGB1 stimulated DCs could activate T cells, DCs stimulated with HMGB1 for 72 h were co-cultivated with naïve T cells derived from rat spleens for 72 h in the presence of Con-A. FACS analyses showed that splenocyte-derived naïve T cells were activated by HMGB1-stimulated DCs indicated by the significantly higher proliferation rate compared with PBS-incubated DCs (**Figure 5A**) (*p* < 0.05). Cytokine analysis of naïve T cells co-incubated with HMGB1-treated DCs revealed that IL-17 and IFN-γ were significantly produced compared with the PBS control (*p* < 0.05), as shown in **Figure 5B**. These results showed that HMGB1 has a significant role in the activation and maturation of DCs, and that HMGB1-activated DCs are a strong stimulator of naïve T cells.

### Blockade of HMGB1 by Glycyrrhizic Acid Alleviates Acute Rejection-Mediated Liver Injury in Recipient Rats

**Figure 6:**
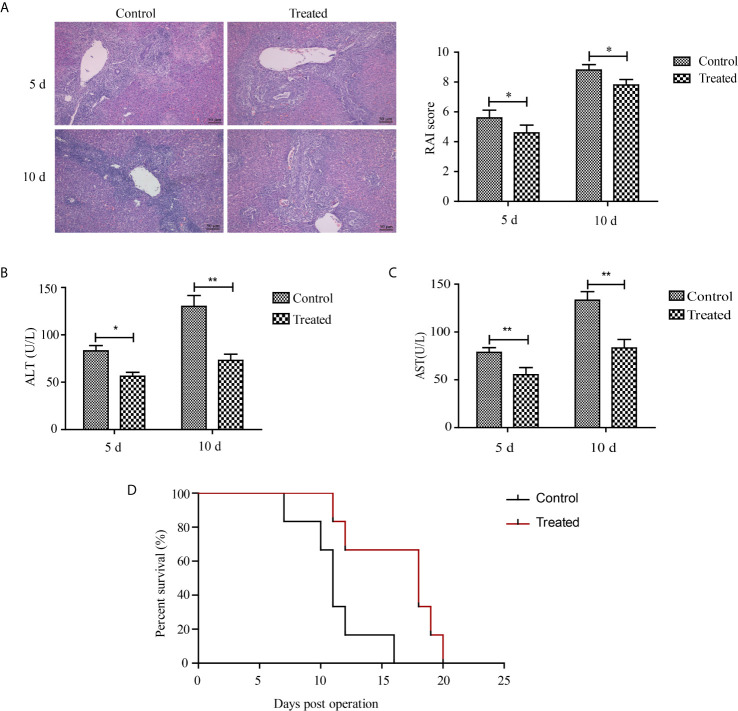
Therapeutic effects of glycyrrhizic acid on liver transplantation. After the donor’s blood was heparinized, livers of the glycyrrhizic acid and control groups were perfused with organ protection solution with or without 200 mg/ml glycyrrhizic acid. **(A)** Representative H&E sections and RAI scores of grafted livers (magnification, ×200). Milder parenchymal necrosis, less inflammatory cell infiltration, and reduced destruction of bile ducts were observed in the glycyrrhizic acid-treated group compared with the control group. **(B, C)** Serum levels of ALT and AST between glycyrrhizic acid-treated group (n=6) and control group. Data are expressed as the mean ± SEM from three independent experiments. *p < 0.05; **p < 0.01. **(D)** Survival curve of recipient rats after liver transplantation. Overall survival was longer in the glycyrrhizic acid group than in the control group. (n=6, median (days): control group vs. treated group = 11 vs. 18, χ^2^ = 6.065, *p* = 0.014).

To identify whether HMGB1 is associated with acute rejection after liver transplantation, livers were perfused with 10 ml ice-cold Ringer’s solution containing 100 units heparin and 200 mg/ml glycyrrhizic acid, a natural HMGB1 blocker, under low pressure after the donor’s blood was heparinized. Representative H&E sections of grafted livers are shown in **Figure 6A**. The glycyrrhizic acid pre-treated livers had mild portal inflammation, reduced destruction of bile ducts, and less inflammatory cell infiltration compared with control livers at 5 and 10 d after transplantation. The RAI scores concurred with the pathological changes. As shown in **Figures 6B, C**, serum levels of ALT and AST in glycyrrhizic acid-treated grafted livers were higher than that in the control group (*p* < 0.05). The mean survival time of the recipient rats was 18 and 11 days for the glycyrrhizic acid-treated group (n=6) and control group (n=6), respectively. The overall survival of the glycyrrhizin group was longer than that of the control group (*p* < 0.05), as shown in **Figure 6D**.

## Discussion

Liver transplantation has therapeutic effects on end-stage liver diseases that are unresponsive to other therapies. Although the liver has tolerogenic potential, the rate of acute rejection is approximately 80% without immunosuppressants; therefore, liver transplants still require immunosuppressive therapies to ensure their long-term survival (5). Complicated cross-talk between DCs and alloreactive T cells is involved in acute liver allograft rejection and is regulated by DAMPs that emerge during ischemia-reperfusion or acute rejection injury (18). The expression pattern and effects of HMGB1, an important DAMP, during acute liver graft rejection are unknown. In this study, we revealed a vital role for HMGB1 in acute rejection after liver transplantation indicating it might be a therapeutic target to avoid inflammatory injury caused by acute rejection.

Acute allograft rejection of liver transplants is characterized by dense inflammatory cell infiltration, hepatocyte injury, portal inflammation, bile duct destruction, and venous inflammation (22). Clinically acute rejection often occurs within the first month, especially 5-7 days after liver transplantation (5). In an orthotopic liver transplantation rat model, severe inflammatory pathological changes occurred in liver grafts of the allogeneic group accompanied by high levels of ALT and AST at 5 and 10 days after transplantation (**Figures 1A, B**). Because T cells and antigen presenting cells (mainly DCs) are involved in the development of acute rejection of solid organ transplants, we quantified CD3^+^ and CD86^+^ cells in liver grafts and found that the allogeneic group had the highest number of T cells and antigen presenting cells, indicating their important roles in liver transplantation (**Figure 1C**). Allograft acute rejection is induced by donor or recipient DCs presenting alloantigens from the graft to the recipient’s T lymphocytes, which can be promoted by the uncontrolled secretion of DAMPs such as HMGB1 (9, 18). MALDI-TOF profiling results showed a higher expression of HMGB1 in the allogeneic group compared with the syngeneic group (**Figure 2**). We observed higher HMGB1 mRNA and protein levels in allograft livers compared with that in sham and syngeneic livers (**Figures 3A, C**). Furthermore, elevated serum levels of HMGB1 detected by ELISA confirmed it was secreted extracellularly where it functioned as an innate immune mediator of liver allograft acute rejection. Our results are in line with previous studies that showed the critical roles of HMGB1 in the acute and chronic allograft rejection of cardiac transplants (24–26).

HMGB1, containing two DNA-binding domains (A-box and B-box) and a highly acidic C-terminal tail, is an important component of innate and adaptive immunity (17). HMGB1 is passively released by necrotic or injured cells and actively secreted from activated immune cells including DCs, macrophages, and natural killer cells (16). HMGB1 enhances innate and adaptive immune responses *via* receptor for advanced glycation endproducts Toll-like receptors 2 and 4 (16). Previous studies revealed HMGB1 was associated with the pathogenesis of inflammatory diseases including systemic lupus erythematosus (27), rheumatoid arthritis (28), and sepsis (29). Because HMGB1 and DCs are important innate-immune mediators, we hypothesized that secreted HMGB1 triggered the activation of DCs and participated in the pathogenesis of the acute allograft rejection of liver transplants. In this study, HMGB1 stimulated rat bone marrow-derived DCs to express CD80, CD86, and MHC II (**Figure 4**). The mature DCs promoted T cell activation determined by CFSE labeling (**Figure 5A**). Our findings are consistent with previous studies demonstrating that full-length HMGB1 or its fragment were endogenous signals for dendritic cell maturation (30, 31). CD4^+^ T cells under certain inflammatory microenvironments containing IFN-γ or IL-6, can be induced into Th1 and Th17 phenotypes, which are essential for inflammatory injury against allografts (9). In our study, a higher percentage of IFN-γ- and IL-17-producing CD4^+^ T cells were observed when naïve CD4^+^ T cells were cultured with HMGB1-pulsed DCs compared with control DCs (**Figure 5B**). IFN-γ and IL-17 also promote a positive feedback loop by triggering the secretion of IFN-γ, IL-6, and other chemokines that facilitate the migration of alloreactive lymphocytes into liver grafts.

HMGB1 promotes the secretion of chemokines and cytokines including IFN-γ, IL-6, and CXCL12, suggesting the inhibition of HMGB1 might prevent immune injury caused by ischemia reperfusion injury or rejection during solid organ transplantation. For example, HMGB1 neutralizing antibodies used as a treatment method for the chronic rejection of heart transplants reduced the number and frequency of CD11b^+^ Ly6C^high^ DCs in recipient spleens (32). Glycyrrhizic acid, a natural blocker of HMGB1 identified by nuclear magnetic resonance analysis, also markedly ameliorated the severity of ischemia reperfusion injury of kidney transplants demonstrated by a decrease in tubular necrosis and neutrophil infiltration (33, 34). Similar to previous studies, the administration of glycyrrhizic acid during donor liver preservation reduced inflammation and hepatocyte damage, and prolonged allograft survival time (**Figure 6**). Glycyrrhizic acid is widely used for the treatment of patients infected with hepatitis C and B and does not inhibit the systemic immune system, suggesting it might be a safe therapeutic agent to prevent the acute allograft rejection of liver transplants (35).

In summary, our results demonstrated the expression characteristics of HMGB1 during the acute rejection of liver transplants. HMGB1 induced the maturation of DCs that polarized naïve CD4^+^ T cells to a Th1 or Th17 phenotype, which promoted immune-related injury. Glycyrrhizic acid, a natural HMGB1 inhibitor, might be a candidate therapeutic agent for HMGB1-related diseases including the acute allograft rejection of liver transplants.

## Data Availability Statement

The raw data supporting the conclusions of this article will be made available by the authors, without undue reservation.

## Ethics Statement

The animal study was reviewed and approved by the Institutional Animal Care and Use Committee of Fujian Medical University.

## Author Contributions

LC and WH conceived and designed the experiments. YC, WZ, and HB performed the experiments. YC, WZ, and HB analyzed the data. YC, LC, and WH wrote the paper. All authors contributed to the article and approved the submitted version.

## Funding

This study was supported by Natural Science Foundation of Fujian Province (Fujian Provincial Natural Science Foundation): (Grant number: 2020J01605, 2020J011076); Fujian Medical Innovation Project: 2020CXA007; The Cultural Project of Innovative Research Team in Fuzhou Health and Family Planning System (Grant number: 2018-S-wt8); Startup Found for scientific research, Fujian Medical University (Grant number: 2019QH1295); High-level hospital foster grants from Fujian Provincial Hospital, Fujian province, China (2019HSJJ08).

## Conflict of Interest

The authors declare that the research was conducted in the absence of any commercial or financial relationships that could be construed as a potential conflict of interest.
